# Modelling the impact of the COVID-19 pandemic on cancer stage migration and excess mortality in Ireland

**DOI:** 10.1016/j.pmedr.2025.103020

**Published:** 2025-03-01

**Authors:** Mengyang Zhang, Paula Tierney, Aline Brennan, Deirdre Murray, Maeve Mullooly, Kathleen Bennett

**Affiliations:** a School of Population Health, RCSI University of Medicine and Health Sciences, Dublin, Ireland; b National Cancer Registry in Ireland, Cork, Ireland; c School of Public Health, University College Cork, Cork, Ireland

**Keywords:** Stage migration, cancer, COVID-19, Pandemic

## Abstract

**Background:**

Cancer treatment services were interrupted during the pandemic, which potentially increased the time to treatment initiation (TTI). This study aimed to model the impact of a hypothetical three- and six-month delay in TTI on stage of breast cancer and non-small cell lung cancer (NSCLC) in Ireland.

**Methods:**

The distribution of cancer stage at diagnosis, net survival at one to five years post diagnosis, and projected cancer incidence for 2020 were obtained for breast cancer and NSCLC, from the National Cancer Registry Ireland. The primary outcome, the probability of an upward stage-shift from stage I to II and stage II to III, is presented with 95 % CIs.

**Results:**

For breast cancer, the stage-shift probability after a hypothetical three-month and six-month delay was 0.13 (0.11, 0.15) and 0.25 (0.21, 0.27) in stage I and 0.09 (0.08, 0.11) and 0.17 (0.14, 0.21) in stage II. For NSCLC, the stage-shift probability after three-month and six-month delays was 0.51 (0.49, 0.53) and 0.76 (0.74, 0.78) in stage I and 0.27 (0.24, 0.30) and 0.47 (0.43, 0.51) in stage II.

**Conclusions:**

The study provides potential evidence for an upward stage migration in those with breast cancer and NSCLC due to the pandemic. It is important to determine the longer-term impacts so that strategies are developed to mitigate adverse effects and improve health system preparedness for future unprecedented events.

## Highlights

• Longer delays in treatment initiation increased the likelihood of stage shift.
• The distribution of stage shift was similar between observed and estimated.
• Prevention of upward stage shift is essential to optimise cancer care and survival.

## 1 Introduction

In response to COVID-19, national restrictions came into place in Ireland on March 27th 2020 including closures to public facilities and a pause in many non-essential healthcare services (O'leary et al., 2021). A considerable reduction in cancer referrals, diagnosis, and treatment was observed in the initial pandemic waves in Ireland (Faculty of Pathology Royal College of Physicians of Ireland et al., 2021). Globally cancer services have been negatively impacted by the pandemic (Angelini et al., 2023; Carvalho et al., 2022). Early evidence from a World Health Organisation report on the impact of COVID-19 on public health services from 194 Ministries of Health showed that more than half of the surveyed countries postponed screening programmes (World Health Organisation, 2020). The disruption to global healthcare services affected not only acute medical care but also elective surgeries due to limited clinical capacity (Glasbey et al., 2021).

For cancer patients, delays in diagnosis and treatment can result in later stage at diagnosis and poorer survival outcomes (Neal et al., 2015). A recent study reported evidence of stage migration during the pandemic period with an increase in the proportion of cancer cases diagnosed at advanced stages and a corresponding decrease in the proportion of early-stage cases in 2020 compared with 2019 levels (Guven et al., 2024). A systematic review and meta-analysis focusing on delays in cancer treatment due to COVID-19 found that a four-week delay in cancer surgery was associated with 6-8 % increased probability of additional death for seven main cancer types including breast and lung cancer (Hanna et al., 2020). Evidence to determine the impact of the pandemic on cancer outcomes is paramount to inform future health policy intervention in the event of further unprecedented disruptions.

However, it is challenging to quantify the COVID-19 impact on cancer stage and health outcomes. Real world evidence can capture patterns for assessing the impact on patient outcomes but can be limited by either data quality or timely availability (Rubinstein et al., 2020). Using near real-time data from the UK DATA-CAN, the one-year total excess deaths ranged from 7165 to 17,910 with 40 % of cancer cases affected by COVID-19 related disruptions in the long term (Lai et al., 2020). Currently data on the long-term effects of health service disruptions on cancer outcomes is not yet available (Riera et al., 2021). The prediction of cumulative excess deaths for breast and colorectal cancer from the US National Cancer Institute suggested 10,000 excess deaths in the next decade, the peak of which would be in 2022 (Sharpless, 2020). The evidence on how disruptions in cancer services due to COVID-19 affected survival outcomes in Ireland is limited (Department of Health et al., 2020).

In this analysis, we focused on the delay in time to treatment initiation (TTI) to estimate the impact of the COVID-19 pandemic on health outcomes for breast and lung cancer. Delays in TTI are defined as delays in the cancer pathway from cancer screening or symptom presentation, GP visit, referral, diagnosis, to first cancer-directed treatment. In Ireland, excluding non-melanoma skin cancer, breast cancer is the most commonly diagnosed cancer and second most common cause of cancer death in women. Non-small cell lung cancer (NSCLC) is the leading cause of cancer death in men and women (National Cancer Registry Ireland, 2023). Breast cancer and NSCLC vary in the stage at diagnosis and survival rates. Breast cancer may be detected symptomatically or by organised screening in Ireland. More screen detected breast cancer cases were diagnosed in earlier stage and five-year survival rate for the period 2014–2018 was 88 % (Brennan et al., 2022). NSCLC, in contrast, is usually symptomatically diagnosed and at a later stage with a lower survival rate (National Cancer Registry Ireland, 2023).

Using the inverse stage-shift model adapted from Degeling et al. (2021) (Degeling et al., 2021) and historical survival data from the National Cancer Registry Ireland (NCRI), we examined the potential impact of delays in TTI due to the COVID-19 pandemic on stage migration and excess mortality for those diagnosed with breast cancer and NSCLC in Ireland in 2020. The first objective was to estimate the probability of stage-shift at early stages of breast and NSCLC after a hypothetical three- and six-month delay in TTI and the proportion of patients that would experience a stage-shift in 2020. The second objective was to quantify the impact on excess cancer-specific mortality by calculating the excess deaths and life years lost (LYL) at five years.

## 2 Methods

### 2.1 Study data

For breast cancer and NSCLC, the stage specific net survival rate at one to five years post-diagnosis, the observed stage distribution in 2019 and 2020, and the projected cancer incidence for 2020 in the absence of the pandemic, were provided by the NCRI. The projected cancer incidence refers to the number of cancer cases that were predicted to be diagnosed in 2020 in the absence of the COVID-19 pandemic. For this analysis, breast cancer was defined as female cancer cases coded as C50 using the International Classification of Disease version 10 (ICD 10) and NSCLC was defined as all (male and female) NSCLC cases coded as ICD-10 C34 excluding all small cell lung cancers and non-staged morphologies. The age-standardised net survival rates were based on 2014–2018 data with follow-up to 2019 (Supplementary Fig. S1). Stage-specific HR between increased TTI and survival for breast cancer and NSCLC (Supplementary Table S1) were taken from the study by Khorana et al. (2019) that investigated the HRs of delays in TTI using data from the US National Cancer Database, which is the most recent published data (Khorana et al., 2019). Because of the shortfall in cancer incidence in 2020, especially shortfalls in early stages, the projected incidence rather than the observed incidence for 2020 was used in the modelling. HR parameters and stage-specific survival rates can be found in Supplementary Material 1.

### 2.2 Stage-shift model

This study used the inverse stage-shift model developed by Degeling et al. (2021) which investigated the impact of delays in TTI on survival outcomes and healthcare costs in Australia (Degeling et al., 2021). This model estimates the stage-shift probability based on the relationship between TTI and mortality rates for specific cancers at different stages. This modelling study focused on stage migration in early stages (stage I and II) due to delays in TTI.

National level data on waiting times for patients from referral to treatment is not available in Ireland. Therefore, hypothetical delays were applied instead of empirical data to measure the impact, based on the known pauses in screening and potential delays in diagnosis and treatment. The baseline scenario refers to no delays in TTI. For stage-shift scenarios, hypothetical delays of three months and six months were selected to represent short and mid-range term delays in TTI. A longer term delay of nine months was also modelled as a sensitivity analysis (Supplementary Material 2). The difference between stage-shift scenarios and the baseline scenario was the impact of the delays in TTI.

This model assumes some patients rather than all patients are influenced by the delay in TTI. The estimation of the stage-shift probability is based on the relationship between TTI and survival rate. The stage-shift occurs if the mortality rate in an early cancer stage is equal to the mortality rate in the next stage. Using the stage-specific HR between increased TTI and survival and the stage specific net survival rate, the stage-shift probability is estimated for a given length of delay. The expected survival in life years was calculated using the Gompertz model for breast cancer and Gamma model for NSCLC. The full model description can be found in Supplementary Material 1.

### 2.3 Outcome variables

Four outcome variables were estimated by the model. The first was the probability of an upward stage migration, which refers to the probability that cancer stage at the time of first cancer-directed treatment progressed to a higher stage due to delays in TTI. The second was the estimated proportion of cases that could undergo a stage-shift. If the distribution of cancer patients is assumed to be the same in each month, the proportion of patients influenced by the delays can be calculated by the stage-shift probability and the length of delays. The third outcome was the estimated excess deaths at five years due to delays in TTI for the projected breast cancer and NSCLC population in 2020, which indicates the difference in survival between the baseline scenario in the absence of the pandemic and the stage-shift scenarios with delays in TTI at the end of year five post-cancer diagnosis. The final outcome was the estimated number of LYL at five years due to delays in TTI, which is defined as the number of expected years of life lost following the upward stage migration at five years post-diagnosis.

### 2.4 Analysis

The observed incidence by cancer stage for breast cancer and NSCLC in 2019 and 2020 was presented to show the variation in the number of cancer cases. The estimated impact on stage-shift probability and the proportion of cases after a three- and six -month delay in TTI were shown by stage. The estimated impact of delays on excess deaths and LYL for the breast cancer and NSCLC population in 2020 was calculated and presented with 95 % CIs.

Based on the new distribution calculated by the stage-shift probability, the observed percentage distribution of cases by cancer stage in 2019 and 2020 and estimated percentage distribution in 2020 were presented as a further comparison of stage migration in early stages. The estimated percentage distribution after a six-month delay was selected for breast cancer and the estimated distribution after a three-month delay was selected for NSCLC.

All analyses were performed using R (version 4.2.2).

### 2.5 Ethics statement

The analysis was conducted using anonymised aggregated data provided by the NCRI for the purpose of research and ethical approval was not required.

## 3 Results

### 3.1 Observed incidence of breast cancer and NSCLC in Ireland in 2019 and 2020 by stage at diagnosis

**Fig. 1:**
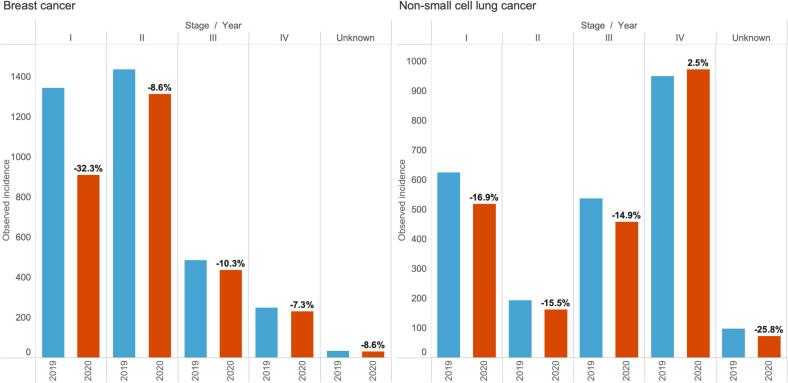
Observed breast cancer and non-small cell lung cancer cases by cancer stage diagnosed in 2019 (blue bar) and 2020 (brown bar) in Ireland. Note: The percentage differences in observed incidence by cancer stage between 2020 and 2019 are shown in labels. (For interpretation of the references to colour in this figure legend, the reader is referred to the web version of this article.)

Fig. 1 shows the observed number of breast cancer and NSCLC cases by stage in 2019 and 2020. Fewer cases were diagnosed in 2020 than in 2019. The overall observed incidence for breast cancer was 2922 in 2020, 17.7 % lower than in 2019. In stage I, 32.3 % fewer cases were diagnosed in 2020 compared with 2019. For NSCLC, the total observed number of cases (*n* = 2187) was 9 % lower than in 2019. There were 16.9 %, 15.5 %, and 14.9 % fewer stage I, II, and III cancers, while there were 2.5 % more cases in stage IV.

### 3.2 Modelling analysis of breast cancer and NSCLC stage migration

Table 1 shows the estimated impact of the hypothetical three- and six-month delay on stage migration for breast cancer and NSCLC in stage I and II and the impact on health outcomes in 2020. A longer-term delay of nine months was also modelled and included in the Supplementary Table S2. The HRs in stage I and II differed, therefore, the stage-shift probability differed by cancer stage. Longer delays in TTI resulted in higher probabilities of stage migration.

**Table 1:** Estimates (95 % CI) of the modelled impact of delays in time to treatment initiation (three and six months) on the stage progression and health outcomes for breast cancer and non-small cell lung cancer (NSCLC).

Estimated impact on stage progression in 2020		Breast cancer		NSCLC	
		Three-month	Six-month	Three -month	Six -month
**Stage I -** **> II**	Probability of stage shift	0.13	0.25	0.51	0.76
		(0.11, 0.15)	(0.21, 0.27)	(0.49, 0.53)	(0.74, 0.78)
	Percentage progressed	3.3 %	12.5 %	12.6 %	37.8 %
		(2.8 %, 3.7 %)	(10.7 %, 13.7 %)	(12.4 %, 13.2 %)	(37.2 %, 38.8 %)
**Stage II -** **> III**	Probability of stage shift	0.09	0.17	0.27	0.47
		(0.08, 0.11)	(0.14, 0.21)	(0.24, 0.30)	(0.43, 0.51)
	Percentage progressed	2.2 %	8.5 %	6.8 %	23.4 %
		(1.9 %, 2.8 %)	(7.2 %, 10.4 %)	(6.0 %, 7.5 %)	(21.3 %, 25.4 %)
		**Breast cancer**		**NSCLC**	
**Estimated impact on health outcomes**		T**hree -month**	S**ix -month**	T**hree -month**	S**ix -month**
**Excess deaths at five years**		8	29	19	57
		(6, 9)	(25, 35)	(18, 19)	(55, 59)
**Life years lost at five years**		20	76	85	260
		(16, 24)	(64, 90)	(81, 89)	(252, 270)

Notes: 95 % CIs in parentheses.

For breast cancer, after a three-month delay in TTI, the stage-shift probability was 0.13 (0.11, 0.15) from stage I to II and 0.09 (0.08, 0.11) from stage II to III, affecting 3.3 % and 2.2 % of breast cancer patients in stage I and II respectively. After a six-month delay in TTI, the stage-shift probability for breast cancer increased to 0.25 (0.21, 0.27) from stage I to II and 0.17 (0.14, 0.21) from stage II to III.

For NSCLC, the stage-shift probability after a three-month delay was 0.51 (0.49, 0.53) for stage I to II and 0.27 (0.24, 0.30) for stage II to III, affecting 12.6 % and 6.8 % of NSCLC patients in stage I and II respectively. After a six-month delay in TTI, the stage-shift probability was 0.76 (0.74, 0.78) from stage I to II and 0.47 (0.43, 0.51) from stage II to III, affecting 37.8 % and 23.4 % of NSCLC patients in stage I and II.

Based on the projected incidence in the absence of the pandemic, the estimated excess deaths at five years for breast cancer was 29 (25, 35) after a six-month TTI delay. The LYL at five years was estimated at 76 (64, 90) after a six-month delay. The estimated impact of delays on NSCLC outcomes was higher. After a six-month delay in TTI, the estimated excess deaths at five years was 57 (55, 59) and LYL at five years was 260 (252, 270).

### 3.3 Observed and estimated breast cancer case distribution by stage

**Fig. 2:**
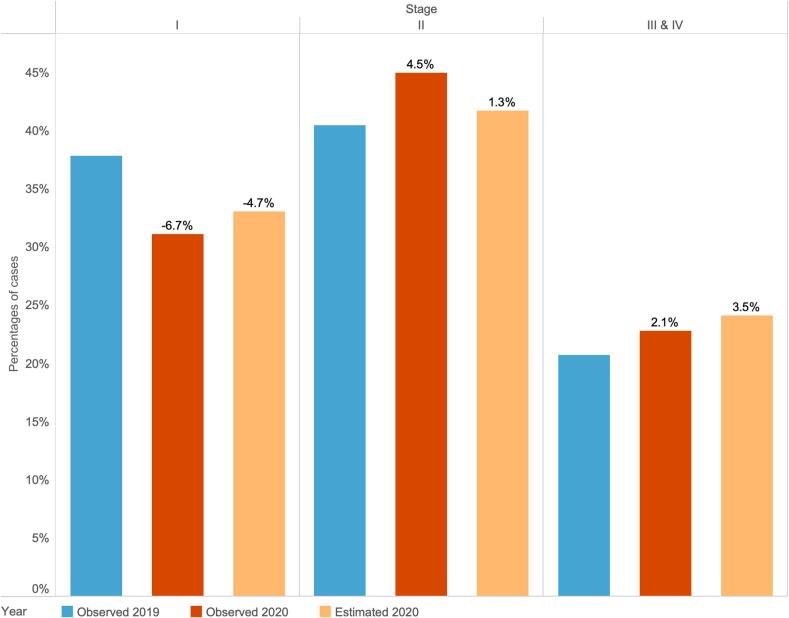
Comparison of proportions of breast cancer cases by cancer stage between observed distributions in 2019 (blue bar), observed distributions in 2020 (brown bar), and estimated distributions in 2020 (light orange bar) after a six-month delay. Note: The percentage differences by cancer stage between observed 2020 and 2019 and between estimated 2020 and observed 2019 are shown in labels. (For interpretation of the references to colour in this figure legend, the reader is referred to the web version of this article.)

Fig. 2 compares the observed percentage distribution of breast cancer cases in 2019 and 2020 with the estimated distribution. Full observed and estimated distribution results by stage and year are shown in Supplementary Table S3. Most breast cancer cases were diagnosed in early stages (stage I and II). The observed and estimated percentage changes corresponded closely with a six-month delay in TTI. There were 6.7 % fewer cancers diagnosed at stage I in 2020 compared with 2019, while there were 4.5 % increased cases in stage II and 2.1 % increased cases in late stages (stage III and IV) in 2020 versus 2019 respectively. The estimated percentage change after a 6-month delay compared with 2019 was −4.7 % in stage I and 1.3 % in stage II, the direction of which was consistent with the observed distribution. However, compared with the observed proportion of cases in 2020, the estimated increase in stage II was lower but the increase in late stages was higher.

### 3.4 Observed and estimated NSCLC case distribution by stage

**Fig. 3:**
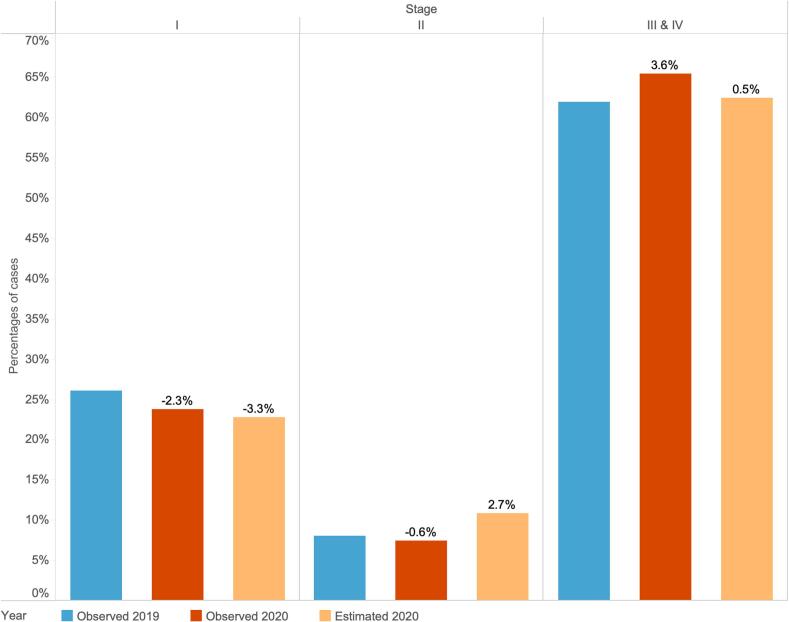
Comparison of proportions of non-small cell lung cancer cases by cancer stage between observed distributions in 2019 (blue bar), observed distributions in 2020 (brown bar), and estimated distributions in 2020 (light orange bar) after a three-month delay. Note: The percentage differences by cancer stage between observed 2020 and 2019 and between estimated 2020 and observed 2019 are shown in labels. (For interpretation of the references to colour in this figure legend, the reader is referred to the web version of this article.)

Fig. 3 compares the changes in stage distribution of NSCLC cases between 2019, 2020 and the modelling estimations. Full observed and estimated distribution results by stage and year are shown in Supplementary Table S3. Unlike breast cancer, most cases were diagnosed at a later stage (stage III and IV). The observed percentage distribution and changes corresponded with a three-month delay in TTI. Compared with 2019, the observed percentage change in stage I and II was −2.3 % and 0.6 % respectively. The estimated percentage change was −3.3 % in stage I and 2.7 % in stage II. There was a higher percentage of cases diagnosed in later stages, especially stage IV, in 2020 compared with 2019.

## 4 Discussion

This modelling study estimated the probability of stage migration, excess deaths, and LYL due to delays in TTI for breast cancer and NSCLC in Ireland. For breast cancer and NSCLC, the model estimated that longer delays in TTI would lead to a higher probability of stage-shift, higher excess deaths, and increased LYL. The model estimated percentage changes in early stages for breast cancer and NSCLC were consistent with the observed percentage changes in cases diagnosed in 2020.

The stage-shift found in early stages for breast cancer and NSCLC were consistent with the findings using the same stage-shift model in other countries (Degeling et al., 2021; Figueroa et al., 2021). The Australian study showed that for breast cancer, the stage-shift probability at stage I was 0.06 after a three-month delay and 0.11 after a six-month delay (Degeling et al., 2021). The findings for lung cancer were 0.08 and 0.16 respectively. These probabilities were lower than our results. A Scottish study estimated the patient distribution assuming stage-shift at all stages after a three-month and six-month delay (Figueroa et al., 2021). Their stage-shift probability at early stages for breast cancer was higher, while the probability for lung cancer was similar (Figueroa et al., 2021). Because the stage-shift probability in this model was determined by the survival rate (HR), and the length of delays, results may not be directly comparable.

A population-based modelling study in England examined excess deaths based on estimated scenarios of delays in cancer referrals from two weeks to six months (Maringe et al., 2020). For breast cancer, this study estimated a 5.5 % increase in excess deaths within one year for up to three month delays and 6.6 % increase within one year for six months delays. For lung cancer, the estimated increase in excess deaths was 7.2 % and 7.7 % respectively. A microsimulation model using the Canadian Cancer Registry data predicted the long-term pandemic impact on cancer mortality due to delays in diagnosis and treatment for 2020 to 2030. The mortality of breast cancer was predicted to increase by 5.9 % resulting in 3116 cumulative excess deaths between 2020 and 2030 (Malagón et al., 2022). For lung cancer, the increase of mortality was 1.1 % and the cumulative excess deaths between 2020 and 2030 was estimated at 3082. Another modelling paper predicted the excess death of breast and lung cancer before and after the COVID-19 in England and found substantial excess deaths in 2020, the impact of which varied by regions and socioeconomic disparities (Arik et al., 2024).

In Ireland, three national level five restrictions were imposed in March, October, and December 2020 in response to the COVID-19 pandemic. Several cancer services were interrupted during the pandemic period, including cancer screening programmes targeted for breast, colorectal and cervical, referral pathways, diagnosis and treatment. Compared with 2019, reduced numbers of women were screened for breast cancer because the breast screening programme was paused between March and October 2020 (National Screening Service, 2020a; National Screening Service, 2020b). Electronic (e)-referrals from GPs to rapid access clinics (RACs) were negatively influenced in the initial pandemic waves (Bambury et al., 2024). There was a significant shortfall in the number of breast e-referrals in March–April and December 2020 and the total number of lung e-referrals in 2020 was 7.4 % lower than the 2019 levels. New attendances at RACs in 2020 were 21 % lower for non-urgent breast clinics and 8.2 % lower for lung clinics compared with 2019 (Faculty of Pathology Royal College of Physicians of Ireland et al., 2021). Significant decreases in cancer surgery, systemic anti-cancer therapy, and radiation oncology activities were observed in 2020 compared with 2019 (Faculty of Pathology Royal College of Physicians of Ireland et al., 2021).

Two hypothetical three-and six-month delays were selected to represent short and mid-range term delays in TTI, in the absence of detailed waiting time data for cancer referral, diagnosis, and treatment in Ireland. In a cohort study estimating the TTI of breast cancer using the data from a primary medical centre and two satellite sites in the US, the average time between cancer presentation and first definitive treatment during the pandemic period was 82 days, or approximately three months (Hawrot et al., 2021). The median TTI of NSCLC for stage I and II was 41 days in the initial period of the pandemic based on the findings from US National Cancer Database (Cone et al., 2020). A longer delay of nine-month was also considered, providing consistent findings.

For breast cancer, the observed percentage change was close to the estimated stage-shift after a six-month delay. Internationally, for cancer types with population-based screening programmes, the disruption in screening resulted in fewer early detections but increased the proportion of cases diagnosed at advanced stages during the pandemic (Guven et al., 2024; Alkatout et al., 2021). Additional evidence from a single healthcare centre in England showed changes in stage distribution of breast cancer at diagnosis (Borsky et al., 2022). They found an increase in patients diagnosed with breast cancer through referral rather than screening due to the restriction policies. They also identified upward stage migration in early stages (from stage 1a to 2a) and increased share of advanced-stage cases (stage III and IV) from 11.2 % in 2019 to 19.6 % in 2020 at initial presentation (Borsky et al., 2022).

For NSCLC, the observed percentage change was close to the estimated stage-shift after a three-month delay and observed proportion of cases in stage I, II and III and stage IV, consistent with published findings (Mynard et al., 2022). Due to COVID-19, the incidence of lung cancer for both males and females in Ireland decreased by 9 % in 2020 compared to 2019 (National Cancer Registry Ireland, 2022). Although lung cancer referral and diagnosis were disrupted, some patients may have been referred to respiratory services via COVID-19 assessment pathways. Therefore, the delays in TTI and stage migration varied by cancer types, which consequently influenced the stage-shift probability and survival.

This study has a number of limitations. The model exclusively focused on early cancer stages. Molecular and pathological differences within stage categories, the estimated impact at later stage cancers, and variations within specific sub-populations were not accounted for. Further, the HRs for the model were based on evidence from the US (Khorana et al., 2019). Although this assumption was required due to limited Irish or other data, it may potentially have introduced uncertainty in results. Cancer progression is influenced by multiple factors and the effect of unmeasured factors cannot be discounted. The duration in delay to TTI may vary from the three- or six-month delay assumed but available cancer waiting time data was unavailable in Ireland. The waiting list numbers published by Irish National Treatment Purchase Fund showed that 40 % of patients were on hospital waiting lists for breast surgery for more than six months in April 2021, which was the most recent data available but only covered data from three hospitals (National Treatment Purchase Fund, 2024). The findings may not be generalizable to other countries with different cancer care pathways and healthcare systems.

## 5 Conclusion

This modelling study suggests increased numbers of excess deaths and LYL, associated with assumed delays in cancer diagnosis and treatment based on estimated probability of stage migration. The provision of cancer services during the pandemic was interrupted, which may have impacted TTI. Prevention of upward stage migration is essential to optimise cancer care and survival. Further validation of this modelling approach is required to investigate the longer-term impact of the COVID-19 pandemic on cancer outcomes. The long-term impact is crucial to develop strategies to mitigate adverse effects and improve health system preparedness for future unprecedented events.

## Funding and Acknowledgment

This work was supported by the 10.13039/501100001593Irish Cancer Society [grant reference number CMP21BEMU]. The opinions, findings and conclusions or recommendations expressed in this material are those of the authors and do not necessarily reflect the view of the Irish Cancer Society. We wish to thank Dr. Joe McDevitt (NCRI) who supported the preparation of cancer survival data in this paper.

## CRediT authorship contribution statement

**Mengyang Zhang:** Writing – review & editing, Writing – original draft, Validation, Methodology, Formal analysis, Conceptualization. **Paula Tierney:** Writing – review & editing, Validation, Data curation. **Aline Brennan:** Writing – review & editing, Validation, Data curation. **Deirdre Murray:** Writing – review & editing, Validation, Supervision, Data curation, Conceptualization. **Maeve Mullooly:** Writing – review & editing, Writing – original draft, Validation, Supervision, Funding acquisition, Conceptualization. **Kathleen Bennett:** Writing – review & editing, Writing – original draft, Supervision, Methodology, Funding acquisition, Conceptualization.

## Declaration of competing interest

The authors declare that they have no known competing financial interests or personal relationships that could have appeared to influence the work reported in this paper.

## Data Availability

Data will be made available on request.

## Appendix A Supplementary data

Supplementary material Image 1
