# Supplementary material for: Modelling the impact of the COVID-19 pandemic on cancer stage migration and excess mortality in Ireland
Source: Prev Med Rep. 2025 Mar 1;52:103020. doi: 10.1016/j.pmedr.2025.103020 (PMC11924935; doi:10.1016/j.pmedr.2025.103020)
Supplement: Supplementary file 1 — Supplementary material [file mmc1.docx]

**Supplementary material 1**

**Overview of the inverse stage-shift model**

This study used the model adapted from the inverse stage-shift model created by Degeling et al for estimating the excess mortality and healthcare cost due to delayed cancer diagnosis and treatment in Australia (1). More details about the model and the R script can be found at: <https://cancerhealthservices.shinyapps.io/oncology_stage_shift/>.

This model assumes some patients rather than all patients are influenced by the delay in time to treatment initiation (TTI) that refers to any delay in any step in the cancer pathway process before the first cancer-direct treatment happens.

The estimation of the stage-shift probability is based on the relationship between TTI and survival rate. Assume the length of delay in TTI, $x$, follows an exponential relation with the event rate, $\lambda$. Then the probability of a patient who occurs a stage migration after a delay can be expressed as:

$$prob\left( x \right)=1-e^{-\lambda x}$$

The hazard ratio (HR) of a delay that results in a stage shift is given as the ratio of stage-specific mortality rate, $r_{j}^{m}$:

$${HR}_{J}^{m}=r_{j+1}^{m}/r_{j}^{m}$$

the stage-specific HR, ${HR}_{j}^{m}$, $j$ is the cancer stage, $m$ indicates mortality. Here a stage-specific 5-year overall survival rate is used to calculate the mortality rate.

Assume the delay in TTI and survival rate follows an exponential relation, the relationship between the expected length of delay that will result in a stage shift and HR of mortality rate can be expressed by the Cox-proportional Hazard Model as follows:

$$\ln\left( {HR}_{J}^{m} \right)=\beta t_{j}^{SS}$$

where the expected length of delay leading to a stage shift, $t_{j}^{SS}$, is the reciprocal of the risk rate, $\lambda$. Khorana et al (2019) investigate the HR of a delay from diagnosis to treatment initiation, ${HR}_{J}^{D}$ ($D$ indicates delays), including surgery, Systemic Anti-Cancer Therapy (SACT), and radiotherapy based on a population-based study using the National Cancer Database from 2004 to 2013 (2), from which the HRs are used for calculating the stage-shift for breast and lung cancer.

Then the risk rate when a stage shift happens equals to:

$$\lambda=\left[ \frac{ln({HR}_{J}^{D})}{t_{j}^{D}} \right]/{\ln\left[ \frac{r_{j+1}^{m}}{r_{j}^{m}} \right]}$$

The estimated excess death and life year lost were calculated based on the non-linear least square regression models. Five models were tested at the beginning: the exponential, Gamma, Gompertz, log-logistic, log-Normal and Weibull. The weighted survival distributions of these models were generated and compared. The Gompertz model and Gamma model were selected for female breast cancer and non-small cell lung cancer (NSCLC) based on the distribution fitness check of quantiles and percentiles of the weighted survival and the distribution plots of historical survival data and estimated distributions in stage-shift scenarios.

**Hazard ratio**

Stage-specific HRs of increased time to treatment initiation (TTI) and stage-specific survival for female breast cancer and NSCLC were taken from the study by Khorana et al (2019) that investigated the HR of a delay in time to treatment initiation based on a population-based study using the National Cancer Database in the US (2). Details of HR parameters by cancer type and stage is shown in Table S1.

**Survival and incidence data**

The cancer- and stage-specific survival rate and the estimated distribution of cancer stage in 2019 for breast cancer and NSCLC in Ireland using data from the National Cancer Registry Ireland (NCRI) is shown in Figure S1. Male breast cancers, all small cell lung cancers, and non-staged morphologies were excluded in the survival and incidence data. Specifically, the age-standardised net survival rate was based on the analysis of 2014-2018 cases with follow up to 2019. For this analysis, breast cancer was defined as female cancer cases coded as C50 using the International Classification of Disease version 10 (ICD 10) and NSCLC was defined as all (male and female) NSCLC cases coded as ICD-10 C34 excluding all small cell lung cancers and non-staged morphologies. The selection was for adult cancer patients aged between 15 and 99. The projected cancer cases for 2020 without the interruption of the pandemic was calculated based on the trend data for 2010-2019. Details of methodology can be found in the NCRI annual report 2023 (3).

The five-year survival rate in stage I was obviously higher than that in stage IV. The survival rate of breast cancer at five years for stage I was 99.1% and the survival rate of stage IV was 32.7%. For NCRI, the survival rate at five years was 57.3% for stage I and 4.2% for stage IV.

**Reference**

1. Degeling K, Baxter NN, Emery J, Jenkins MA, Franchini F, Gibbs P, et al. An inverse stage-shift model to estimate the excess mortality and health economic impact of delayed access to cancer services due to the COVID‐19 pandemic. Asia-Pacific Journal of Clinical Oncology. 2021.

2. Khorana AA, Tullio K, Elson P, Pennell NA, Grobmyer SR, Kalady MF, et al. Time to initial cancer treatment in the United States and association with survival over time: an observational study. PloS one. 2019;14(3):e0213209.

3. National Cancer Registry Ireland. Cancer in Ireland 1994-2021: Annual statistical report of the National Cancer Registry. Cork, Ireland: National Cancer Registry Ireland; 2023.

**Table S1: Hazard ratios parameters used in the inverse stage progression model**

| Cancer | Stage I | 95% CI | Stage II | 95% CI |
| --- | --- | --- | --- | --- |
| Female breast | 1.018 | (1.015–1.020) | 1.012 | (1.010–1.015) |
| Non-small cell lung cancer | 1.032 | (1.031–1.034) | 1.016 | (1.014–1.018) |

**Figure S1: Age-standardised net survival of cancer patients in Ireland for diagnosis years 2014-2018 with follow up to 2019**


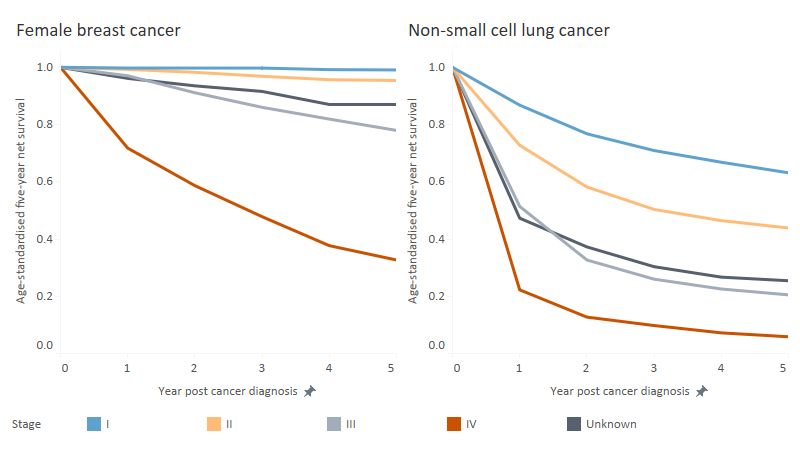


Note: The survival rates are cumulative net survival rates. Male breast cancers and small cell lung cancers are excluded from the survival figures.

**Supplementary material 2**

Except the three-month and six-month delays in TTI, a longer term delay of nine months was also modelled. The result of the estimated impact on stage migration for breast cancer and NSCLC in stage I and II and the estimated impact on health outcomes for the cancer population in 2020 is shown in Table S2.

As to the observed and estimated distribution by stage for breast cancer and NSCLC, the comparison of the observed percentage distribution of cancer cases in 2019 and 2020 with the estimated distribution in 2020 after three, six, and nine months delays in TTI is shown in Table S3.

**Table S2: Stage-shift modelling result with a nine-month delay in time to treatment initiation for breast cancer and non-small cell lung cancer (NSCLC)**

| **Estimated impact on stage progression in 2020** | | | |
| --- | --- | --- | --- |
|  |  | **Breast cancer** | **NSCLC** |
| Stage I -> II | Probability of stage shift | 0.35 | 0.88 |
|  |  | (0.30, 0.38) | (0.87, 0.89) |
|  | Percentage progressed | 26.3% | 65.9% |
|  |  | (22.7%, 28.5%) | (65.3%, 67.0%) |
| Stage II -> III | Probability of stage shift | 0.24 | 0.61 |
|  |  | (0.21, 0.30) | (0.56, 0.66) |
|  | Percentage progressed | 18.4% | 46.0% |
|  |  | (15.7%, 22.2%) | (42.3%, 49.2%) |
| **Estimated impact on health outcomes (stage migration in stage I and II**) | | | |
|  |  | **Breast cancer** | **NSCLC** |
| Excess deaths at five years | | 62 | 101 |
|  |  | (55, 73) | (99, 104) |
| Life year lost at five years | | 162 | 464 |
|  |  | (138, 190) | (453, 477) |

**Table S3: Observed number of cases and the distribution by cancer stage in 2020, the difference in cancer stage distribution between 2019 and 2020, and the estimated percentage change in stage I and II after three-month, six-month, and nine-month delays in time to treatment initiation**

| **Female breast cancer** | |  |  |  |  |  |  |  |  |
| --- | --- | --- | --- | --- | --- | --- | --- | --- | --- |
|  |  |  |  |  | **Estimated 2020** |  | **Estimated 2020 - Observed 2019** | | |
| **Stage** | **Observed 2019** | **Observed 2020** | **Observed 2020-2019** | **three-month** | **six-month** | **nine-month** | **three-month** | **six-month** | **nine-month** |
| **I** | 37.8% | 31.1% | -6.7% | 36.6% | 33.1% | 27.9% | -1.3% | -4.7% | -9.9% |
|  |  |  |  | (36.4%, 36.8%) | (32.7%, 33.8%) | (27.0%, 29.3%) |  |  |  |
| **II** | 40.5% | 45.0% | 4.5% | 40.8% | 41.8% | 43.0% | 0.4% | 1.3% | 2.5% |
|  |  |  |  | (40.8%, 40.8%) | (41.4%, 41.6%) | (42.3%, 42.7%) |  |  |  |
| **III + IV** | 20.7% | 22.8% | 2.1% | 21.6% | 24.2% | 28.1% | 0.9% | 3.5% | 7.4% |
|  |  |  |  | (21.5%, 21.8%) | (23.6%, 24.9%) | (27.0%, 29.7%) |  |  |  |
| **Non-small cell lung cancer** | | |  |  |  |  |  |  |  |
|  |  |  |  |  | **Estimated 2020** |  | **Estimated 2020 - Observed 2019** | | |
| **Stage** | **Observed 2019** | **Observed 2020** | **Observed 2020-2019** | **three-month** | **six-month** | **nine-month** | **three-month** | **six-month** | **nine-month** |
| **I** | 26.0% | 23.8% | -2.3% | 22.7% | 16.2% | 8.9% | -3.3% | -9.8% | -17.2% |
|  |  |  |  | (22.6%, 22.8%) | (15.9%, 16.3%) | (8.6%, 9.0%) |  |  |  |
| **II** | 8.0% | 7.5% | -0.6% | 10.8% | 16.0% | 21.5% | 2.7% | 8.0% | 13.5% |
|  |  |  |  | (10.9%, 10.8%) | (16.1%, 16.0%) | (21.5%, 21.6%) |  |  |  |
| **III + IV** | 61.9% | 65.5% | 3.6% | 62.4% | 63.8% | 65.6% | 0.5% | 1.9% | 3.7% |
|  |  |  |  | (62.4%, 62.5%) | (63.6%, 63.6%) | (65.3%, 65.8%) |  |  |  |

Notes: 95% CIs in parentheses.
